# A Surgeon Involved in Basic Research - on the Occasion of Studying Abroad

**DOI:** 10.14789/jmj.JMJ23-0027-R

**Published:** 2023-11-29

**Authors:** TOMOAKI ITO

**Affiliations:** 1 Department of Surgery, Juntendo University Shizuoka Hospital, Juntendo University School of Medicine, Shizuoka, Japan; 1 Department of Surgery, Juntendo University Shizuoka Hospital, Juntendo University School of Medicine, Shizuoka, Japan

**Keywords:** ERC/mesothelin, fatty acid synthase, gastric cancer, maternal stress, lung tumor

## Abstract

I, the author, expressed gratitude for receiving the 45^th^ Juntendo Medical School Alumni Association Academic Encouragement Award in May 2023. After completing medical school and surgical training at Juntendo University, I embarked on a new challenge by pursuing a Ph.D. in basic clinical research, with a focus on gastric cancer, the third leading cause of cancer related death in Japan. Collaborating with various experts, I obtained a Ph.D. in cancer research studies in 2014. Subsequently, I pursued further research opportunities in the United States, where I undertook multiple projects focusing on cancer and maternal stress. I would like to present several studies, ERC/mesothelin, fatty acid synthase, and maternal stress in this manuscript.

On returning to clinical practice at Juntendo University Shizuoka Hospital in 2019, I developed an interest in various clinical issues and decided to address these through experiments. In collaborating with several researchers at the Shizuoka Medical Research Center for Disasters, our ongoing research aims to answer several clinical questions.

Furthermore, I aspire to guide junior staff in the future and am grateful for the invaluable connections and opportunities provided by Juntendo University.

## Introduction

I (the author) received the 45^th^ Juntendo Medical School Alumni Association Academic Encouragement Award in May 2023. I would like to thank all concerned persons. After graduating from Juntendo University School of Medicine in 2000, I trained in the Department of Surgery at Juntendo University, led by Professor Masahiko Tsurumaru, until 2008. In 2009, I was transferred to the Department of Surgery, led by Professor Koichi Sato, at Juntendo University Shizuoka Hospital, where I currently work. At the time of my transfer to Juntendo Shizuoka Hospital, I had been a surgeon for 10 years, so I decided to take on a new challenge and began my path to obtain a Ph.D. degree in basic research in addition to my clinical work.

Malignant tumors are the leading cause of death in Japan. The second most common is heart disease, followed by senility. Among the malignant tumors, gastric cancer is the third leading cause of death in Japan.

Although the incidence of gastric cancer has decreased due to the decrease of *Helicobacter pylori* infection and improvement of dietary habits, I have researched gastric cancer. I was introduced to Dr. Kazunori Kajino and Dr. Masaaki Abe under the supervision of Professor Okio Hino of the Department of Pathology and Oncology. In 2011, I began my research while attending the main hospital once a week. They taught me how to conduct the experiments and helped me a lot.

## ERC/mesothelin is expressed in human gastric cancer tissues and cell lines^1)^

**Figure 1:**
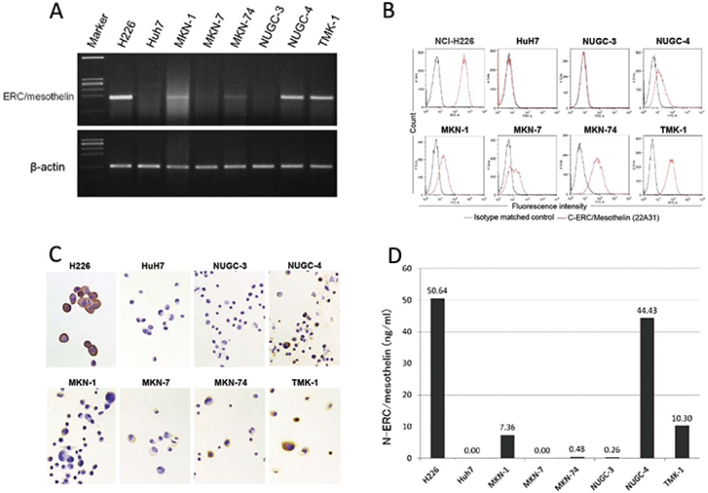
ERC expression^1)^. (A) ERC/mesothelin transcript was detected in human gastric cancer cell lines by RT-PCR. H226 cells were used as a positive control. Huh7 cells were used as a negative control. The other cell lines are derived from human gastric cancer. (B) Cell surface C-ERC/mesothelin expression in the human gastric cancer cell lines as determined by flow cytometry. (C) Immunohistochemical staining of C-ERC/mesothelin in the human gastric cancer cell lines using 22A31 antibody. Magnification, x400. (D) ELISA detected N-ERC/mesothelin that had been secreted into the cell culture media of human gastric cancer cell lines.

ERC/mesothelin, a protein found in mesothelioma and other malignancies, is encoded by the ERC/mesothelin gene (*MSLN*), which produces a 71-kDa precursor protein. This precursor protein undergoes cleavage, resulting in the formation of a 31-kDa N-terminal protein (N-ERC/mesothelin) and a 40-kDa C-terminal protein (C-ERC/mesothelin). N-ERC/mesothelin is a soluble protein that has been identified as a diagnostic serum marker for mesothelioma and ovarian cancer. Although C-ERC/mesothelin is expressed in gastric cancer tissues, the diagnostic significance of serum N-ERC levels in gastric cancer remains unexplored. This study aimed to address this knowledge gap by investigating the importance of serum N-ERC levels in the diagnosis of gastric cancer and exploring C-ERC/mesothelin expression in human gastric cancer tissues and cell lines. C-ERC/mesothelin expression was examined through immunohistochemistry in tissue samples from 50 gastric cancer patients, and C-ERC/mesothelin expression in six gastric cancer cell lines (MKN-1, MKN-7, MKN-74, NUGC-3, NUGC-4, and TMK-1) was assessed using various techniques such as reverse transcription- polymerase chain reaction (RT-PCR), flow cytometry, immunohistochemistry, and immunoblotting. Additionally, N-ERC/mesothelin concentrations in cultured cell supernatants and sera from patients with gastric cancer were measured using an enzyme-linked immunosorbent assay (ELISA). RT-PCR, flow cytometry, and immunohistochemistry confirmed the presence of ERC/mesothelin mRNA and C-ERC protein in five of the six gastric cancer cell lines investigated (MKN-1, MKN-7, MKN-74, NUGC-4, and TMK-1) (Figure 1A-C). ELISA detected N-ERC/mesothelin in the supernatants of the three gastric cancer cell lines (MKN-1, NUGC-4, and TMK-1) (Figure 1D). However, the concentration of N-ERC/mesothelin in the sera of gastric cancer patients was comparable to that observed in the sera of normal controls (data not shown). Furthermore, C-ERC/mesothelin expression is associated with the lymphatic invasion of gastric cancer tissues. Although N-ERC/mesothelin is secreted by gastric cancer cell lines, it does not appear to be a useful serum marker for gastric cancer.

## Elevated levels of serum fatty acid synthase in patients with gastric carcinoma^2)^

**Figure 2:**
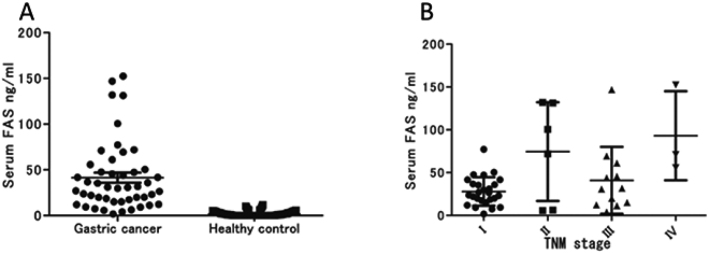
FAS levels^2)^.(A) Serum FAS levels of gastric cancer patients were significantly higher than healthy controls (P<0.0001; Mann-Whitney U test). (B) When the TNM staging was analyzed, there was no significance in serum FAS concentration (P=0.0603; Kruskal‐Wallis test). TNM, tumor-node-metastasis; FAS, fatty acid synthase.

In order to facilitate optimal treatment strategies, the development of biomarkers for early detection and timely intervention of new cancers, as well as for recurrent cases, is crucial. Fatty acid synthase (FAS) is highly expressed in various human cancers, and is a potential biomarker. However, the applicability of FAS for the detection of gastric cancer remains unexplored. In this study, we conducted an initial evaluation of serum FAS levels as markers of gastric carcinoma. This study included 47 patients diagnosed with gastric cancer and 150 healthy individuals as controls. Blood samples were collected from each patient prior to treatment, and serum FAS levels were quantified using an ELISA, allowing for a comparison between the two groups. The analysis revealed significantly higher serum FAS levels in patients with gastric cancer (95% confidence interval [CI]: 30.37-52.46) compared to the healthy controls (95% CI: 1.331-2.131) (Figure 2A). Importantly, elevated FAS levels were observed in patients with early stage tumors (Figure 2B). These findings highlighted the potential of serum FAS as a biomarker for sensitive and specific detection of gastric cancer.

## Research in the USA

I received my Ph.D. in Research Studies in 2014. As I became involved in these basic studies, coupled with my long-held desire to study, I met Professor Malcolm Brock and Dr. Kathleen Gabrielson at Johns Hopkins University, United States. I told them that I wanted to conduct research in the United States, and they accepted my request. In February 2016, I began my long-term study abroad experience. In the United States, there are very few patients with gastric cancer, and it is no exaggeration to say that none of them are interested in gastric cancer. Therefore, I began several research projects simultaneously instead of focusing on only one project. One was on maternal stress, funded by the MEXT*-Supported Program for the Strategic Research Foundation at Private Universities (*Ministry of Education, Culture, Sports, Science, and Technology), 2015-2019 at Shizuoka Medical Research Center for Disaster.

## Prenatal stress enhances NNK-induced lung tumors in A/J mice^3)^

**Figure 3:**
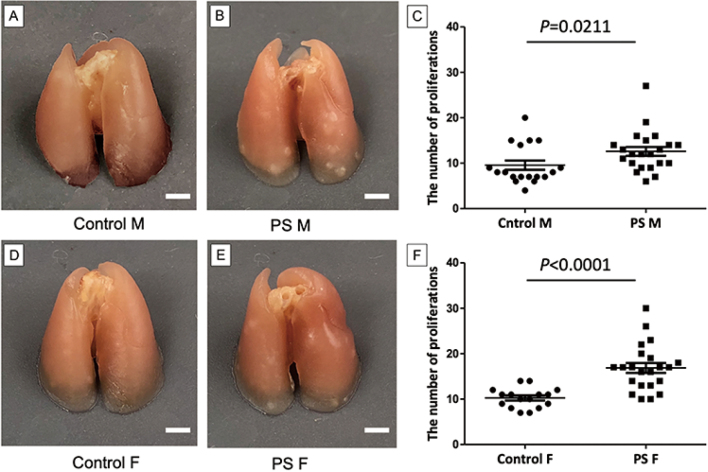
Macroscopic comparison of control and PS group lungs^3)^. PS group had significantly more proliferations than control group (males and females). M, Male; F, Female; PS, Prenatal stress.

Offspring born to mothers who experience stress during pregnancy have an elevated risk of cancer later in life. However, to the best of our knowledge, no animal studies have investigated this association. In this study, we examined whether prenatal stress (PS) in A/J mice influences the development of lung tumors following exposure to tobacco-specific nitrosamine 4-(methylnitrosamino)-1-(3-pyridyl)-1-butanone (NNK). Timed-bred A/J mice were randomly assigned on gestation day 12.5 to either PS, achieved through restraint for five consecutive days, or a control group without restraint. Adult offspring from both control and stressed pregnancies were administered three injections of NNK (50 mg/kg every other day) and euthanized 16 weeks later for lung examination. Compared to the control group, dams subjected to PS exhibited significantly elevated levels of plasma corticosterone, increased adrenal weights, and reduced fetal weights without any fetal loss. Prenatally stressed litters experienced a notably higher neonatal mortality rate within the first week of life, and the surviving male and female offspring demonstrated increased lung epithelial proliferation (Figure 3), as well as increased tumor multiplicity, larger tumor area, and more aggressive tumor morphology. PS also resulted in the presence of advanced atypical adenomatous hyperplastic lesions. While we observed no difference in NNK-derived methyl DNA adducts in the lungs, PS treatment led to a significant increase in the infiltration of CD3+ and Foxp3+ T cells into lung tumors. PS significantly enhanced tumor multiplicity, tumor area, and tumor morphology in NNK-induced lung tumors. PS did not affect the production of NNK-derived methyl DNA adducts but increased lymphocytic infiltration into lung tumors. To the best of our knowledge, this is the first animal model of PS that is used to assess the development of cancer in offspring. This novel mouse model holds potential for further elucidation of the mechanisms underlying the enhancement of carcinogenesis by PS.

## PS enhances atherosclerosis and telomere shortening in ApoE knockout mouse offspring^4)^

**Figure 4:**
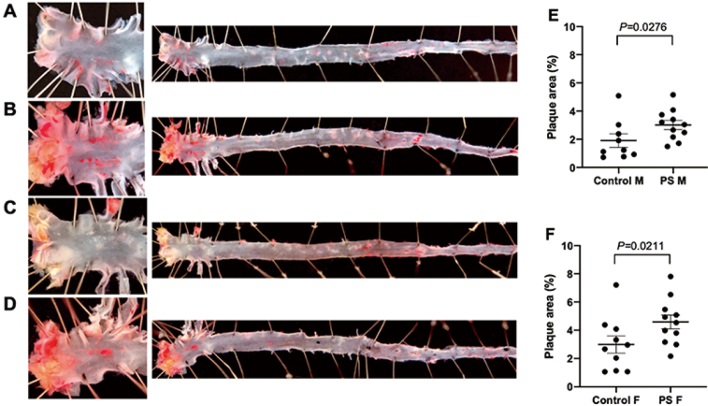
Comparison between plaque area in the whole aorta of the control group and prenatal stress group who were euthanized at 25 wk^4)^. PS group had significantly more plaques than the control group in both of male and female. M, Male; F, Female; PS, Prenatal stress.

Offspring born to mothers experiencing stress during pregnancy face an elevated risk of atherosclerosis later in life; however, only a limited number of animal models have explored the underlying mechanisms of this phenomenon. In the present study, time-bred ApoE-knockout mice were used to investigate this phenomenon. Pregnancy in mice was confirmed via ultrasound, and on gestation day 8.5, mice were randomly assigned to either a control group (no stress) or a PS group, which involved subjecting the mice to 2 h of restraint for five consecutive days. The PS group showed a significant increase in plasma corticosterone levels during pregnancy. Neonatal mortality within the first week of life was higher in the litters of PS mice. After euthanasia, adult offspring from the PS group at 25 weeks of age had significantly higher body weights than those from the control group. Adult offspring were serially imaged using ultrasound to measure plaque thickness and were subjected to macroscopic and microscopic examinations of plaque pathology. The PS group displayed increased plaque thickness, as determined using ultrasonography, gross evaluation (Figure 4), and histological analysis, along with heightened infiltration of macrophages into the aortic root and valve at 25 weeks. At 5 weeks of age, mice in the PS group exhibited a significant decrease in mean arterial pressure, but blood pressure levels normalized by 10 weeks. Considering that the PS-induced increases in apoptosis and telomere shortening are stress-sensitive, telomere lengths were compared in the aorta of 10-week-old mice. The telomeres were significantly shorter in the PS group than in the control group. These findings encourage further exploration of how stress affects telomere shortening in animal models and the human aorta. Moreover, this model provides a valuable platform for investigating the roles of PS, telomere biology, and the pathogenesis of atherosclerosis in adults.

## Perspectives and Significance

The physical and mental health consequences of stress in adults are well-recognized; however, the impact of stress on unborn children during pregnancy is more challenging to comprehend. However, recent epidemiological investigations have documented detrimental health effects in populations exposed to PS caused by genocides, natural disasters, pandemics, and wars. The mechanisms underlying these effects, which likely persist across generations, remain largely unexplored. This study aimed to investigate the acceleration of atherosclerosis and lung tumors due to PS, with a focus on understanding the associated mechanisms. Animal models offer a valuable approach for examining these mechanisms, providing insights that can be extrapolated to human populations to develop preventive or mitigating strategies^4)^.

## After returning to Japan

I started my practical work at Juntendo University Shizuoka Hospital in May 2019, after returning from the United States. This marked the 20th year since I became a doctor. Although my clinical specialty is the gastrointestinal tract, I received a grant from KAKENHI and I am currently continuing my research.

When I returned to clinical practice, I became interested in the various clinical issues that I faced and wondered whether I could clarify them through experiments.

With the cooperation of several researchers at the Shizuoka Medical Research Center for Disasters, our ongoing research aims to answer several clinical questions.

In the future, I would like to guide junior staff so that they can understand the enjoyment of basic research. I can also reaffirm that this is because of the solid and broad connections I have established at Juntendo during my research. I would like to thank you for the 45^th^ Juntendo Medical School Alumni Association Academic Encouragement Award.

## Funding

The studies were supported in part by a Grant-in-Aid from MEXT- Supported Program for the Strategic Research Foundation at Private Universities, 2015-19 and grants from the Vehicle Racing Commemorative Foundation.

## Author contributions

Not applicable.

## Conflicts of interest statement

The author declare that there are no conflicts of interest.
